# The Utility of the Cachexia Index and the Modified Glasgow Score in Young Patients With Breast Cancer

**DOI:** 10.7759/cureus.59301

**Published:** 2024-04-29

**Authors:** Ismail Beypinar, Hacer Demir, Yaşar Culha, Furkan Kaya

**Affiliations:** 1 Medical Oncology, Alanya Alaaddin Keykubat University, Antalya, TUR; 2 Medical Oncology, Afyonkarahisar Health Sciences University, Afyonkarahisar, TUR; 3 Radiology, Afyonkarahisar Health Sciences University, Afyonkarahisar, TUR

**Keywords:** prognosis, nutrition, young breast cancer, breast cancer, modified glasgow prognostic score, cancer cachexia index

## Abstract

Background

Breast cancer is the most common cancer in women. Body composition and inflammatory markers are increasingly important for predicting cancer prognosis. The Cancer Cachexia Index (CXI) and the modified Glasgow Prognostic Score (GPS) are two new markers evaluating prognosis in cancer. In this study, we evaluated the utility of the CXI and the modified GPS in young patients with breast cancer.

Methods

Eighty patients diagnosed between 2012 and 2023 were included in the study. The following information was recorded: patient features, pathological subtype, estrogen receptor and human epidermal growth factor receptor-2 (HER-2) status, disease stage, therapies, disease recurrence, and last control or death date. The CXI and the modified GPS were calculated using clinical data, including skeletal muscle index, albumin, C-reactive protein, and neutrophil-to-lymphocyte ratio.

Results

There were no differences in overall survival with respect to the CXI in the study population (p=0.96). Only stage 4 patients showed statistically significant survival differences according to the CXI (p=0.046). Although the median survival time was not reached for the modified GPS groups, there was a statistical overall survival difference favoring the negative group (p=0.017). No significant differences were observed in disease-free survival due to the CXI (p=0.128). In multivariate analysis, no factors, including the modified GPS and the CXI, influenced overall survival. There was a significant effect of the modified GPS and body mass index on recurrence (p=0.037; p=0.034). The CXI had a non-significant marginal p-value (p=0.074).

Conclusion

Our study showed that the modified GPS may be related to disease-free survival and overall survival, whereas the CXI has a more prominent prognostic effect on overall survival in advanced-stage breast cancers. In early-stage and young patients, optimization of risk scores is lacking.

## Introduction

Breast cancer is the most common cancer in women [1,2]. Recent data confirm the decline in mortality from this disease by more than 2% in developed countries [3]. In contrast to the decrease in mortality in both developed and developing countries, breast cancer continues to have the highest incidence [2]. Despite population scanning programs, effective treatment options, and precision medicine, high rates of local and distant metastasis are still observed in breast cancer [4]. Although standardized medicinal approaches are applied to all patients, different outcomes are generally observed. Disease heterogeneity and individualized treatment options are key to success. Emerging biomarkers are very important for the stratification of patients and for foreseeing disease outcomes [5]. Disease progression in cancer is confirmed to be related to the tumor, treatment, and host. Tumor type, stage, grade, treatment, and nutritional status are some of these factors [6-8]. There is still an unidentified complex mechanism between nutrition and tumorigenesis [9]. In the past, serum albumin and body mass index (BMI) were commonly used to evaluate the nutritional health of cancer patients. However, BMI and albumin have several limitations as indicators of nutritional status. Firstly, as people age, their proportion of body fat increases while their muscle mass declines; nevertheless, these changes may not be reflected by changes in their height, weight, or BMI, making the specificity of BMI weak [10,11]. Secondly, patients with liver cirrhosis demonstrate post-translational alterations to albumin that limit its amount and function. Many liver-related disorders, such as liver cirrhosis, impact the level and function of albumin [12]. There is also a prominent difference in body composition between the sexes. Males tend to have more muscle tissue while females have more fat tissue, affecting both healthy populations and cancer patients [13-15].

Obesity is a risk factor for breast cancer. Additionally, disease recurrence, drug resistance, and decreased survival rates are associated with obesity. High BMI has been shown to relate to decreased response to neoadjuvant therapy [16-18]. The term "sarcopenic obesity" is defined in patients with high BMI and low muscle volume, which masks malnutrition. In these patients, malnutrition cannot be assessed with only BMI, making other clinical tools essential [19,20]. Multiple studies in different cancer types have shown that clinical and nutritional tools are independent prognostic factors. Glasgow Prognostic Score (GPS), neutrophil-lymphocyte ratio (NLR), prognostic nutritional index (PNI), and platelet-lymphocyte ratio (PLR) predict cancer-specific survival in various disease types [21,22].

The cachexia index (CXI) is a recent entity that combines nutritional and immune status, including the skeletal muscle index (SMI), albumin, and NLR. It may show a better cachectic status than the individual tools in cancer patients. CXI is confirmed to have a prognostic effect on non-Hodgkin lymphoma and advanced lung cancer [23-26]. Different cancers and different age groups still need investigation. In this study, we evaluated the utility of CXI and mGPS in young patients with breast cancer.

## Materials and methods

Patients

A retrospective analysis of breast cancer cases diagnosed between 2012 and 2023 was conducted at the oncology division of Afyonkarahisar Health Sciences University. The study included patients who were under 40 years of age. The following information was logged: patient features, pathological subtype, estrogen receptor (ER) and human epidermal growth factor receptor-2 (HER-2) status, disease stage, therapy options, disease recurrence, and last control or death date. Patients over 40 years at the time of diagnosis and those lacking adequate imaging, laboratory, and follow-up data were excluded. Only patients between 18 and 40 years old with complete clinical data and high-quality computed tomography (CT) images were included in the study. Eighty patients in the CXI group and 76 patients in the mGPS group were evaluated. Data for four patients could not be assessed for mGPS.

Body composition assessment, CT analysis, and biochemical calculation

Total skeletal muscle area (SMA) was determined from axial CT scans that included all abdominal muscles (psoas, erector spinae, quadratus lumborum, external and internal oblique, and rectus abdominis) [27]. Data were collected from electronic medical records encompassing clinical, laboratory, and radiological perspectives. Serum albumin level (g/dL), multiplied by the SMI and NLR, was used to determine the CXI. We measured the SMI using a technique previously established, focusing on the pectoralis major and minor muscles [23,26].

Statistics

IBM SPSS Statistics for Windows, Version 25 (Released 2017; IBM Corp., Armonk, New York) was used for statistical analysis. Descriptive data for continuous variables were displayed as means or medians, while frequencies and percentages were provided for categorical variables. The Pearson chi-squared test was utilized to evaluate connections between categorical variables. The Z-test was used to observe differences within the subgroups of the chi-square test. Using the Kaplan-Meier product-limit approach, OS curves were calculated. Life tables were created to facilitate the appropriate survival analysis. The study population was evaluated using three cutoff values. Receiver operating characteristic (ROC) analysis was used to select the best cutoff value, though no optimal cutoff value was observed. Two cutoff values were derived from the literature; the first was 16.58, determined from studies evaluating lymphoma and lung cancer [26]. The second cutoff value for the CXI was 53, from the hepatocellular carcinoma study [28]. Despite the absence of up-to-date literature on breast cancer patients, the median CXI value of 77.2 was used as the cutoff for our study population. This median cutoff value was employed for statistical analysis. None of the patients had a score of 2 in the mGPS, which was evaluated as positive or negative.

Ethics

The Afyonkarahisar Health Sciences University Faculty of Medicine's ethical committee approved the study, which was conducted in accordance with the tenets of the Declaration of Helsinki and all relevant laws.

## Results

Comparative patient characteristics

Eighty patients were included in the study. The median patient age was 36 years. All the patients were female; no male patients were diagnosed before the age of 40 in our archives. Seventy-five patients had invasive ductal carcinoma, while five patients had other histologic subtypes (one medullary carcinoma, one lobular carcinoma, and mixed histologic subtypes). Cancer was localized in the right breast for 41 patients, while 39 patients had left-sided disease. Thirty-nine patients underwent total mastectomy, and 38 underwent breast-conserving surgery. Three patients had no surgery. Sixty-four patients were ER-positive, and 20 exhibited HER-2 amplification. The number of patients by stages I, II, III, and IV was 25, 29, 19, and 7, respectively. Seventy-three patients received adjuvant chemotherapy, while 61 received radiation therapy in an adjuvant setting. Only seven patients had stage 4 disease at the time of diagnosis. Twelve patients experienced disease recurrence at a median follow-up of 39 months. Seventeen patients had positive mGPS scores, while 59 had negative scores. The characteristics of the study population, according to CXI and mGPS, are described in Table 1.

**Table 1:** The comparative data of the study population according to CXI and mGPS. *Statistical difference in subgroup analysis. CXI: cachexia index; mGPS: modified Glasgow Prognostic Index; BMI: body mass index; Adj: adjuvant; CHT: chemotherapy; RT: radiotherapy; BCS: breast-conserving surgery; ER: estrogen receptor; HER-2: human epidermal growth receptor-2.

Number of patients (n)	Low CXI (n)	High CXI (n)	p-value	Negative mGPS (n)	High mGPS (n)	p-value
Age (median)	36	36	0.79	35	38	<0.001
Smoking			0.33			0.27
No	38	38		55	17	
Yes	3	1		4	0	
BMI kg/m^2^ (median)	27.3	26.8	0.82	26.7	32.2	0.054
Stage			0.11			0.28
I	14	11		22	3	
II	14	15		18	9	
III	12	7		14	3	
IV	1*	6*	0.040*	5	2	
Adj CHT			0.041			0.17
None	1	6		4	3	
Received	40	33		55	14	
Adj RT			0.95			0.011
None	9	9		10	8	
Received	31	30		48	9	
Surgery type			0.06			0.07
None	0*	5*	0.02*	3	2	
Mastectomy	21	17		24	11	
BCS	20	17		32*	4*	0.025*
CXI	N/A	N/A	N/A	79.1	63	0.054
mGPS			0.071	N/A	N/A	N/A
Negative	27	32				
Positive	12	5				
ER			0.91			0.65
Negative	8	8		11	4	
Positive	33	31		48	13	
HER-2			0.24			0.42
Negative	33	27		43	14	
Positive	8	12		16	3	
Side			0.65			0.69
Right	21	18		28	9	
Left	20	21		31	8	
Recurrence			0.31			0.14
No	31	30		48	10	
Yes	8	4		7	4	

Survival outcomes

**Figure 1:**
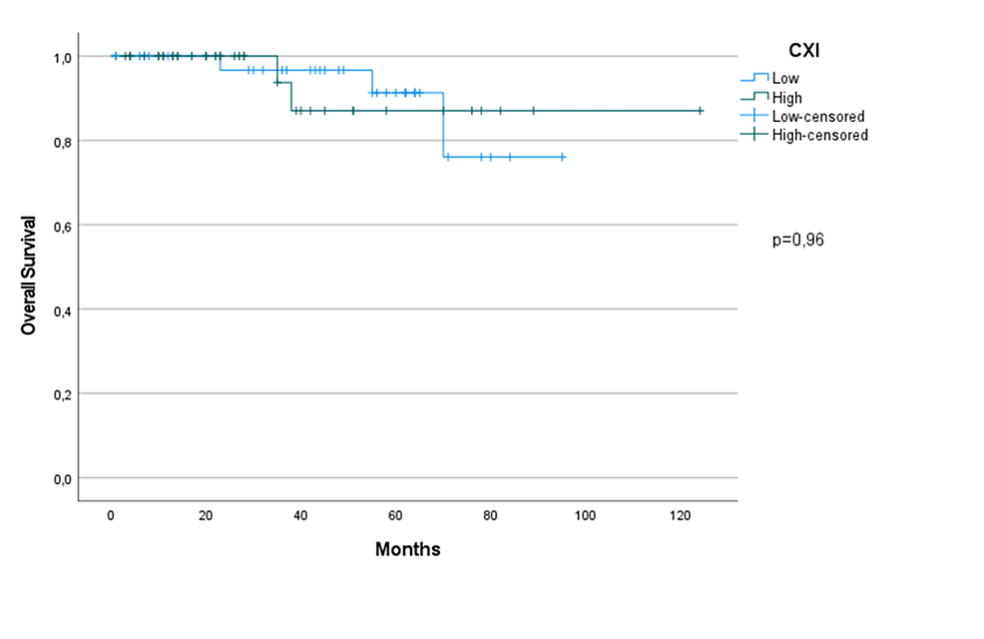
Overall survival of the study population in terms of CXI. CXI: cachexia index.

**Figure 2:**
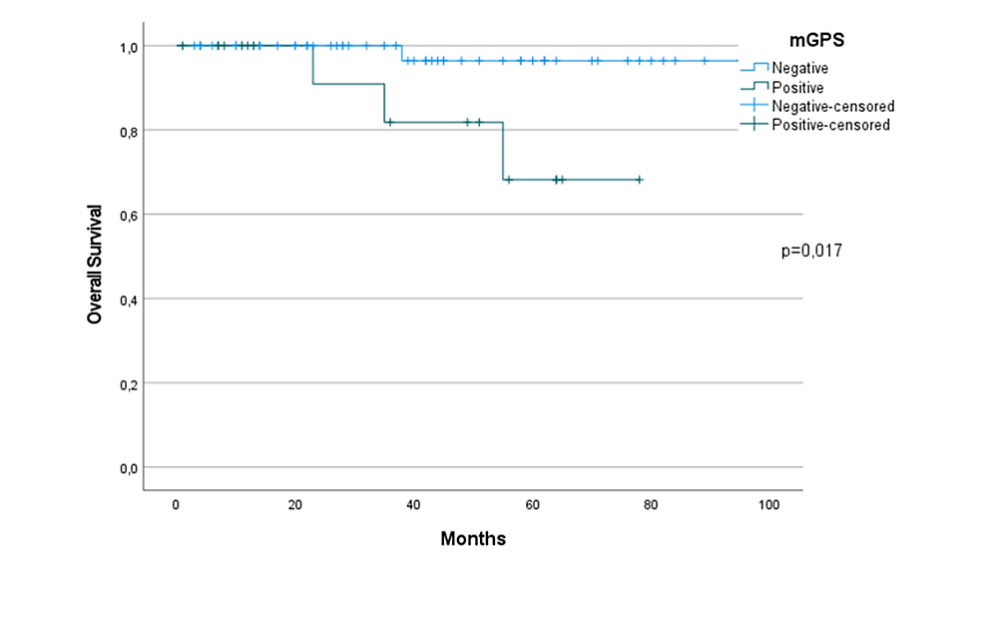
Overall survival of the study population in terms of mGPS. mGPS: modified Glasgow Prognostic Index.

There were no differences in OS in terms of the CXI in the study population (p=0.96) (Figure 1). Neither group reached the median survival time. Only stage four patients showed statistically significant survival differences according to CXI (p=0.046) (Figure 2). The median OS in the low CXI group was 23 months, but was not reached in the high CXI group. Although the median survival time was not reached for the mGPS groups, there was a statistical OS difference favoring the negative group (p=0.017). No difference was observed in the OS for mGPS in terms of disease stage. In the univariate analysis, stage, ER positivity, and HER-2 positivity were not related to OS. Surgery type, adjuvant RT, and adjuvant chemotherapy had statistically significant effects on OS (p<0.001, p=0.039, and p=0.004, respectively).

There was no significant difference in DFS due to CXI (p=0.128). Also, mGPS had no predictive effect on recurrence (p=0.85). Smoking, adjuvant RT, and surgery type had a predictive effect on recurrence (p=0.052, p=0.006, p=0.017 respectively). Stage, ER, HER-2, and adjuvant chemotherapy had no effect on disease relapse.

In multivariate analysis, no factors, including mGPS and CXI, influenced OS. There was a significant effect of mGPS and BMI on tumor recurrence (p=0.037, p=0.034) (Table 2). CXI had a non-significant marginal p-value (p=0.074).

**Table 2:** The multivariate analysis of the study population in terms of OS and DFS. CXI: cachexia index; mGPS: modified Glasgow Prognostic Index; BMI: body mass index; Adj: adjuvant; CHT: chemotherapy; RT: radiotherapy; ER: estrogen receptor; HER-2: human epidermal growth receptor-2, HR: hazard ratio; OS: overall survival; DFS: disease-free survival.

Variables	OS		DFS	
	HR	p-value	HR	p-value
Age	0.52	0.83	51.6	0.057
Smoking	7.07	0.96	-	0.11
BMI	2.1	0.64	22	0.034
mGPS	-	0.73	-	0.037
CXI	0.23	0.96	-	0.074
ER	-	0.90	-	-
HER-2	-	0.92	-	0.44
Surgery type	-	0.86	-	0.13
Adj CHT	-	0.97	-	-
Adj RT	-	0.74	-	-

## Discussion

In this study, we found that mGPS may be a better predictor of OS than CXI in young breast cancer patients. In contrast, CXI may have a better predictive effect on recurrence than mGPS. To our current knowledge, this is the first study to investigate CXI and mGPS in young breast cancer patients.

The prognostic effect of CXI in metastatic settings has been observed in several cancer types. In terms of breast cancer, young patients have not been evaluated for CXI, and no standardized cutoff has been determined. Some studies have utilized the same value for different diseases such as lung cancer and lymphomas [26], while some researchers used different cutoff values, such as for hepatocellular carcinoma [28]. When our study population was categorized according to the studies by Go et al., only two patients were included in the low CXI group, which made comparison difficult. Without available literature on breast cancer, our study used its own cutoff value of 77.2, which is much higher than those used in other studies. The main reason for the high cutoff level may be related to the young patient age with high muscle volume and good nutrition status. Early cancers and young patients may have different nutritional statuses compared to older and advanced-stage patients. CXI score may be a vital option for patients with underlying hepatic dysfunction to strengthen the nutritional status behind albumin [28]. Although prior studies demonstrated sarcopenic conditions in young patients, more prominent markers for malnutrition and tumor cachexia are still needed [29]. The study evaluating early-stage gastric cancer in 175 patients who underwent curative surgery demonstrates the utility of CXI. A low CXI proved to be an independent prognostic factor for both OS and DFS [30]. Gong et al. also confirmed that CXI has a prognostic effect on gastric cancer [31]. Another study investigating the utility of CXI in resectable pancreatic cancer showed the prognostic effect of CXI for both OS and DFS [32]. In a large retrospective study investigating the utility of CXI in colorectal cancer, high CXI was found to be related to better OS. In this study, no difference was observed in DFS due to CXI levels [33]. Although these studies investigate early-stage diseases like our study, these study populations are mainly composed of older patients. The other difference from our study is the high recurrence and death rates of gastric and pancreatic cancers compared to breast cancer. Especially in gastrointestinal cancers, tumor cachexia is a more common phenomenon even in early stages than in breast cancer.

In a large retrospective study investigating the nutritional status of breast cancer via albumin and BMI composing the Nutritional Risk Index (NRI), decreased NRI was confirmed to be related to worse OS in early breast cancer [34]. In our study, we could not demonstrate any nutritional parameter related to OS. The main reason for this phenomenon may be the small sample size and low recurrence and death rates.

In metastatic breast cancer patients treated with eribulin, comparing mGPS and PNI, low PNI and positive mGPS were shown to be related to TTF in univariate analysis. Low PNI was proven to have an effect on OS and TTF in multivariate analysis [35]. In contrast to our study, this study comprised metastatic patients. In a large retrospective case study investigating metastatic prostate cancer, mGPS was confirmed to be an independent factor for OS [36]. Also, mGPS was prognostic for head and neck cancers. Nakayama et al. suggested a scoring system for routine practice [37]. These studies confirm the strong prognostic effect of mGPS especially in metastatic carcinoma, but evidence for early-stage cancer is still lacking. Our study evaluated early-stage and young breast cancers for further evaluation of the mGPS score in breast cancer. There may be different cutoffs for young patients with breast cancer in terms of CXI.

Limitations

Our study had a small sample size with a low incidence of death and recurrence, which made statistical analysis challenging. The cutoff values were difficult to determine due to the study population's features. mGPS could not be categorized into three groups because of the evaluated markers.

## Conclusions

Comparing mGPS and CXI in young breast cancer was the main objective of our study. According to our study's results, mGPS may be related to DFS and OS, while CXI has a more prominent prognostic effect on OS in advanced-stage breast cancers. It is difficult to generalize our findings due to the heterogeneous and small study population. Further studies evaluating young breast cancer for body composition and inflammatory markers are needed in both early and advanced stages to confirm our results.
